# Enhanced production of nitric oxide, reactive oxygen species, and pro-inflammatory cytokines in very long chain saturated fatty acid-accumulated macrophages

**DOI:** 10.1186/1476-511X-7-48

**Published:** 2008-11-28

**Authors:** Naotake Yanagisawa, Kazunori Shimada, Tetsuro Miyazaki, Atsumi Kume, Yohei Kitamura, Katsuhiko Sumiyoshi, Takashi Kiyanagi, Takafumi Iesaki, Nao Inoue, Hiroyuki Daida

**Affiliations:** 1 Department of Cardiovascular Medicine, Juntendo University School of Medicine, Tokyo, Japan; 2 Nutritional Science Institute, Morinaga Milk Industry Co., Ltd., Kanagawa, Japan; 3 Department of Organ and Cell Physiology, Juntendo University School of Medicine, Tokyo, Japan

## Abstract

**Background:**

Deterioration of peroxisomal β-oxidation activity causes an accumulation of very long chain saturated fatty acids (VLCSFA) in various organs. We have recently reported that the levels of VLCSFA in the plasma and/or membranes of blood cells were significantly higher in patients with metabolic syndrome and in patients with coronary artery disease than the controls. The aim of the present study is to investigate the effect of VLCSFA accumulation on inflammatory and oxidative responses in VLCSFA-accumulated macrophages derived from X-linked adrenoleukodystrophy (X-ALD) protein (ALDP)-deficient mice.

**Results:**

Elevated levels of VLCSFA were confirmed in macrophages from ALDP-deficient mice. The levels of nitric oxide (NO) production stimulated by lipopolysaccharide (LPS) and interferon-γ (IFN-γ), intracellular reactive oxygen species (ROS), and pro-inflammatory cytokines, including tumor necrosis factor-α (TNF-α), interluekin-6 (IL-6), and interleukin-12p70 (IL-12p70), were significantly higher in macrophages from ALDP-deficient mice than in those from wild-type mice. The inducible NO synthase (iNOS) mRNA expression also showed an increase in macrophages from ALDP-deficient mice.

**Conclusion:**

These results suggested that VLCSFA accumulation in macrophages may contribute to the pathogenesis of inflammatory diseases through the enhancement of inflammatory and oxidative responses.

## Background

Deterioration in peroxisomal β-oxidation activity plays a critical role in ageing- and inflammatory-related disorders through the accumulation of very long chain saturated fatty acids (VLCSFA) in various organs [1,2]. VLCSFA with more than 22 carbon atoms are almost exclusively oxidized in peroxisomes [3]. Accumulation of VLCSFA is reported to be associated with increased membrane microviscosity and membrane disorders [4,5]. Indeed, hexacosanoic acid (C26:0), a VLCSFA, is accumulated in plasma, the membranes of blood cells, and/or tissue of patients with X-ALD, which is characterized by progressive demyelination and adrenal insufficiency [6,7].

In patients with X-ALD, the expression of pro-inflammatory cytokines, including tumor necrosis factor-α (TNF-α) and interleukin-1β (IL-1β), in activated macrophages and astrocytes was up-regulated in brain lesions [8]. As well as increased expression of inducible nitric oxide synthesis (iNOS), pro-inflammatory cytokines, such as TNF-α, IL1-β, and IL-6, were observed in astrocytes and microglia [9,10]. Furthermore, TNF-α production in peripheral blood mononuclear cells (PBMC) after lipopolysaccharide (LPS) administration was significantly higher in X-ALD patients than in controls [11,12]. The production of NO and superoxide anion (O_2_^-^) after treatment with LPS and oxidized low-density lipoprotein (OxLDL) was increased in C26:0-enriched rat glial cells [13]. These results suggested that VLCSFA accumulation in various cells including macrophages, microglia, and astrocytes from X-linked ALD is associated with enhanced inflammatory responses.

Monocytes/macrophages also play a key role in the process of atherosclerotic diseases [14,15]. Activated macrophages in atherosclerotic lesions produce a wide variety of mediators, including NO [16], reactive oxygen species (ROS) [17], and pro-inflammatory cytokines [18]. We recently reported that C26:0 is accumulated in the plasma and membranes of blood cells in patients with metabolic syndrome and in patients with coronary artery disease [19,20]. These results suggest that VLCSFA accumulation may play an important role in lifestyle related diseases, such as metabolic syndrome and atherosclerotic diseases; however, the precise mechanism by which VLCSFA accumulation contributes to the initiation and progression of such diseases is uncertain. Therefore, the present study was conducted to investigate the effect of endogenous VLCSFA accumulation on inflammatory and oxidative responses in macrophages derived from ALDP-deficient mice [21].

## Methods

### Reagents

RPMI1640 media, fetal bovine serum (FBS), penicillin, streptomycin, phosphate buffered saline without calcium and magnesium (PBS (-)), and Hank's balanced salt solution (HBSS) were purchased from Gibco (Carlsbad, CA, USA). Thioglycollate was obtained from Becton Dickinson (Cockeysville, MD, USA). Phorbol myristate acetate (PMA), dimethyl sulfoxide (DMSO), 2',7'-dichlorofluorescin diacetate (DCFH-DA), *Escherichia coli *lipopolysaccharide (LPS, 0111:B4), sulfanilamide, naphthylethylenediamine dihydrochloride, phosphoric acid (H_3_PO_4_), sodium nitrite (NaNO_2_), boron trifluoride methanol, nonacosanoic acid, and tricosenoic acid were purchased from Sigma-Aldrich (St. Louis, MO, USA). Murine recombinant interferon (IFN)-γ was purchased from BD Pharmingen (San Diego, CA, USA).

### Mice

ALDP-deficient mice [21] backcrossed to C57BL/6J for 10 generations were obtained from the National Institute for Longevity Sciences of the National Center for Geriatrics and Gerontology (Obu City, Aichi, Japan) and males aged 12 to 14 weeks were used for experiments. Male C57BL/6J, wild-type counterparts, were purchased from Charles River Laboratory Inc. (Japan). All animals were housed and maintained in specific pathogen-free condition at a controlled temperature (23 ± 2°C), on a 12 h light/dark cycle, and fed a normal chow diet (Funabashi Farm, Chiba, Japan) and tap water *ad libitum*. The animal experiments were approved by the Morinaga Milk Industry Animal Research Committee, and the mice were maintained according to the guides for the care and use of laboratory animals of Morinaga Milk Industry Co., Ltd.

### Cell Culture

To isolate thioglycollate-elicited murine peritoneal exudate macrophages (MPMs), mice were injected intraperitoneally with 2 ml of 4% (wt/vol) thioglycollate solution. Four days later, they were sacrificed by anesthetizing with ether and their abdominal cavities washed out twice with 5 ml of cold PBS (-). The peritoneal lavage fluid was centrifuged at 1200 rpm, 4°C for 10 min. After washing twice, cell pellets were gently resuspended at 1 × 10^6 ^cells/ml in RPMI1640 medium with 2 mM L-glutamine, supplemented with 10% endotoxin-free FBS, 100 U/ml penicillin and 100 μg/ml streptomycin. To recover adherent cells, MPMs were incubated for 2 h and washed with PBS(-) twice, and non-adherent cells were removed by aspiration. All culture incubations were performed in a humidified 37°C, 5% CO_2 _incubator unless otherwise stated. Cell viability was checked using Tripan blue exclusion and the viability was greater than 95%.

### Measurements of Plasma Lipid and Glucose Levels

Mice were fasted for 16 h and blood samples were harvested by cardiac puncture after ether anesthesia and collected into EDTA-disodium containing tubes. Samples were centrifuged at 3000 rpm for 15 min to obtain plasma. Plasma lipid such as triglyceride (TG), total cholesterol (TC), very low-density lipoprotein cholesterol (VLDL-C), low-density lipoprotein cholesterol (LDL-C), and high-density lipoprotein cholesterol (HDL-C) were measured by the HPLC column method at Skylite Biotech Inc. (Akita, Japan) [22], and plasma glucose levels were measured by the Glucose CII Test Wako (Wako Pure Chemical Industries, Ltd, Tokyo, Japan).

### C26:0 Quantification and Fatty Acid Composition Analyses

MPMs (1.5 × 10^7 ^cells) were rinsed with PBS(-) and total lipid was extracted by the Folch method [23]. The extract was then transmethylated with 14% boron trifluoride methanol solution at 90°C for 90 min. Quantification of C22:0 and C26:0 was performed with a gas chromatography-mass spectrometry (GS-MS) system (QP2010, Shimadzu Corporation, Kyoto, Japan) equipped with a fused silica capillary column (Rtx-5 MS, 30 m × 0.25 mm i.d.; 0.25 μm film thickness, Restek, USA) using nonacosanoic acid (C29:0) methyl ester as an internal standard. The mass spectrum acquisitions of C22:0, C26:0, and C29:0 methyl esters were performed in selected-ion monitoring (SIM) mode and the target ions of these fatty acid methyl esters were 354.50, 410.40, and 452.40 m/z, respectively. For fatty acid composition analysis, total lipid extract from MPMs was transmethylated as described above and incubated at 90°C for 60 min. The measurement of fatty acids was performed with a GC-FID system (6890N, Agilent technologies, Tokyo, Japan) equipped with a fused silica capillary column (Omegamax 250, 30 m × 0.25 mm i.d.; 0.25 μm film thickness, Supelco, USA) using tricosenoic acid (C23:0) methyl ester as an internal standard.

### Nitrite Assay

The production of NO was determined by Griess reagent solution [24]. MPMs (1.0 × 10^5 ^cells/well) were seeded in a 96-well flat-bottom plates and stimulated with various concentrations (1, 10, 100 ng/ml) of LPS plus 2 ng/ml IFN-γ for 24 h for dose-dependent analysis. Control media contained neither LPS nor IFN-γ. For time-dependent analysis, MPMs were stimulated with 10 ng/ml of LPS plus 2 ng/ml IFN-γ and incubated for various times (0, 12, 18, 24 h). After incubation, 50 μl of cell culture supernatant was mixed with 100 μl of Greiss reagent solution (1% sulfanilamide and 0.1% naphthylethylenediamine dihydrochloride in 2.5% H_3_PO_4_, respectively) and incubated for 10 min at room temperature. Absorbance of the mixture was measured at 550 nm in a microplate reader (Corona, Ibaraki, Japan) and NO concentration was determined using a serial dilution (1 to 125 μM final concentrations) of NaNO_2 _in culture medium as a standard. The levels of NO production were normalized to cell viablitiy using CellTiter-Glo Luminescent Cell Viability Assay (Promega, WI, USA).

### Real-time Quantitative Reverse Transcription-PCR for iNOS

MPMs (1.0 × 10^6 ^cells/well) were seeded in 6-well flat-bottom plates and stimulated with 10 ng/ml LPS plus 2 ng/ml IFN-γ for 18 hours. Then, total RNA was extracted using Trizol (Invitrogen, CA, USA) following the manufacturer's protocol. Total RNA (40 ng/each reaction) was reverse transcribed into cDNA using random primers (High Capacity cDNA Reverse Transcription kit; Applied Biosystems, CA, USA) at 25°C for 10 min, 37°C for 120 min, and 85°C for 5 s. For PCR amplification, 4 μl of reverse transcription reaction was added to a 16 μl reaction mixture containing TaqMan Fast Universal PCR Master Mix (Applied Biosystems, CA, USA) and TaqMan Gene Expression Assays reagents (Applied Biosystems, CA, USA) for iNOS (Mm00440485_m1). The β-actin gene (Mm00607939_s1) was amplified in the same experiment to serve as the reference gene. The reaction was carried out in an Applied Biosystems 7500 Fast Real-Time PCR System at 95°C for 20 s, cycled at 95°C for 3 s and 60°C for 30 s for 40 cycles. The mRNA expression levels were normalized to those of β-actin.

### Intracellular Reactive Oxygen Species (ROS) Assay

To measure ROS levels in MPMs, flow cytometric analysis was conducted with DCFH-DA [25]. MPMs (1.0 × 10^6 ^cells/well) were seeded in 6-well flat-bottom plates then incubated with 10 μM of DCFH-DA in PBS(-) for 15 min at 37°C. After incubation, MPMs were rinsed with PBS(-) to remove unincorporated DCFH-DA and then stimulated with 0.5 μg/ml of PMA for 20 min at 37°C. To stop reaction, MPMs were rinsed with PBS(-) and gently scraped with a cell scraper. Cell suspensions were transferred to a tube and washed twice with cold PBS(-). Cellular fluorescence was determined using a FACSCanto flow cytometer (BD Biosciences, USA). Intracellular fluorescence was measured at 530/30 nm after excitation of cells at 488 nm with an argon ion laser. MPMs were discerned by the combination of forward-scattered and side-scattered laser light and aggregated and/or fragmented cells were excluded. 10,000 events were recorded for analyses.

### Quantification of Pro-inflammatory Cytokine Production

MPMs (1.0 × 10^5 ^cells/well) were seeded in 96-well flat-bottom plates and stimulated with 10 ng/ml LPS plus 2 ng/ml of IFN-γ for 24 h. Culture supernatants were collected and cytokines (IL-6, TNF-α and IL-12p70) in media were measured by flow cytometer using the cytometric bead array (CBA) method (Mouse inflammation kit; BD Biosciences, USA) according to the manufacturer's instructions. Data were acquired using a FACS Canto flow cytometer (BD Biosciences, USA) and analyzed using BD cytometric bead array software (BD Biosciences, USA). The levels of pro-inflammatory cytokines production were normalized to cell viability using the CellTiter-Glo Luminescent Cell Viability Assay (Promega, WI, USA).

### Statistical Analysis

All values are expressed as mean ± SD. Statistical analysis was performed with a two-sided Student's t-test using SAS software (version 9.1.3; SAS Institute, Cary, NC). Differences were considered significant at *P *< 0.05.

## Results

### Body Weight, Plasma Lipid, and Glucose Levels in Mice

Table 1 shows levels of body weight, plasma lipids, and glucose in each group. Average body weight for ALDP-deficient mice was significantly higher than that for wild-type mice (*P *< 0.05). The levels of plasma TG, TC, VLDL-C, LDL-C, HDL-C, or glucose were not significantly different between the two groups.

**Table 1:** Body weight, plasma lipid, and glucose levels in mice

	Wild-type	ALDP-deficient
Body weight (g)	25.8 ± 1.8	28.2 ± 2.4*
TG (mg/dL)	49.9 ± 13.6	57.9 ± 10.1
TC (mg/dL)	93.3 ± 5.8	95.7 ± 6.8
VLDL-C (mg/dL)	4.53 ± 1.0	4.80 ± 1.1
LDL-C (mg/dL)	10.7 ± 1.1	10.3 ± 1.3
HDL-C (mg/dL)	77.9 ± 5.0	80.5 ± 7.7
Glucose (mg/dL)	214 ± 77	213 ± 57

Data represents mean ± SD, n = 10. ALDP, adrenoleukodystrophy protein; TG, triglyceride; TC, total cholesterol; VLDL-C, very low-density lipoprotein cholesterol, LDL-C, low-density lipoprotein cholesterol, HDL-C, high-density lipoprotein cholesterol. *P < 0.05 for comparison with wild-type.

### Quantification of C26:0

**Figure 1:**
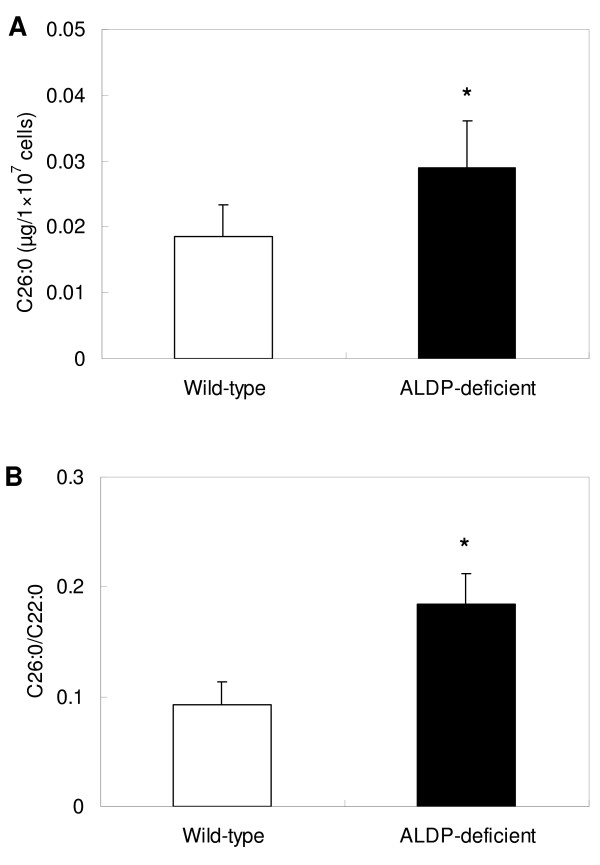
**Levels of C26:0 in macrophages**. Total lipid was extracted from macrophages using the Folch method, and C26:0 and C22:0 were measured with the GC-MS system, adding C29:0 as an internal standard. (A) Concentrations of C26:0 in macrophages. (B) C26:0/C22:0 ratio in macrophages. Results are expressed as mean ± SD, n = 8. * *P *< 0.05 for comparison with wild-type.

The concentration of C26:0 and the ratio of C26:0/C22:0 in MPMs are shown in Fig. 1A and 1B, respectively. Both C26:0 concentrations and C26:0/C22:0 in MPMs from ALDP-deficient mice were significantly higher compared with those of wild-type mice (*P *< 0.05 and *P *< 0.01, respectively).

### Fatty Acid Composition of MPMs

Fatty acid composition in MPMs is shown in Table 2. There was no significant difference in relative percentage of total saturated fatty acids (SFA) between the two groups. However, MPMs from ALDP-deficient mice had a significantly lower relative percentage of behenic acid (C22:0) (*P *< 0.01) and a significantly higher relative percentage of lignoceric acid (C24:0) (*P *< 0.01) than those from wild-type mice. The relative percentage of total n-6 polyunsaturated fatty acid (n-6 PUFA) was similar between the two groups. However, the relative percentage of linoleic acid (LA: C18:2n-6) was significantly higher than that of wild-type mice. Although, there were no significant differences in n-3 PUFA or n-6 PUFA between the two groups, the ratio of n-3/n-6 was significantly lower in ALDP-deficient mice than in wild-type mice (*P *< 0.05).

**Table 2:** Fatty acid composition in macrophages

Fatty acids	Wild-type (% of total lipid)	ALDP-deficient (% of total lipid)
C14:0	1.08 ± 0.21	1.18 ± 0.26
C16:0	21.27 ± 0.96	20.77 ± 0.62
C16:1n-7	2.66 ± 0.24	2.82 ± 0.56
C18:0	14.37 ± 0.47	14.65 ± 1.19
C18:1n-9	9.45 ± 0.80	9.74 ± 0.61
C18:2n-6	11.65 ± 0.27	12.52 ± 0.60**
C18:3n-3	0.27 ± 0.09	0.26 ± 0.06
C20:0	0.27 ± 0.13	0.32 ± 0.11
C20:3n-6	1.37 ± 0.16	1.40 ± 0.14
C20:4n-6	9.82 ± 0.35	9.31 ± 0.52
C20:5n-3	0.34 ± 0.05	0.34 ± 0.06
C22:0	0.40 ± 0.04	0.34 ± 0.04**
C22:4n-6	2.68 ± 0.13	2.54 ± 0.18
C22:5n-3	2.67 ± 0.30	2.45 ± 0.18
C24:0	0.59 ± 0.05	0.69 ± 0.06**
C22:6n-3	4.44 ± 0.40	4.16 ± 0.27
SFA	37.98 ± 1.18	37.94 ± 0.91
n-6 PUFA	25.52 ± 0.69	25.77 ± 0.74
n-3 PUFA	7.73 ± 0.51	7.21 ± 0.38
n-3/n-6	0.30 ± 0.02	0.28 ± 0.01*
n-3/AA	0.79 ± 0.04	0.78 ± 0.06

Data represents mean ± SD, n = 8. ALDP, adrenoleukodystrophy protein; SFA, saturated fatty acid; PUFA, polyunsaturated fatty acid; AA, arachidonic acid. *P < 0.05, **P < 0.01 for comparison with wild-type.

### Dose- and Time-Dependent NO Production and iNOS Gene Expression

**Figure 2:**
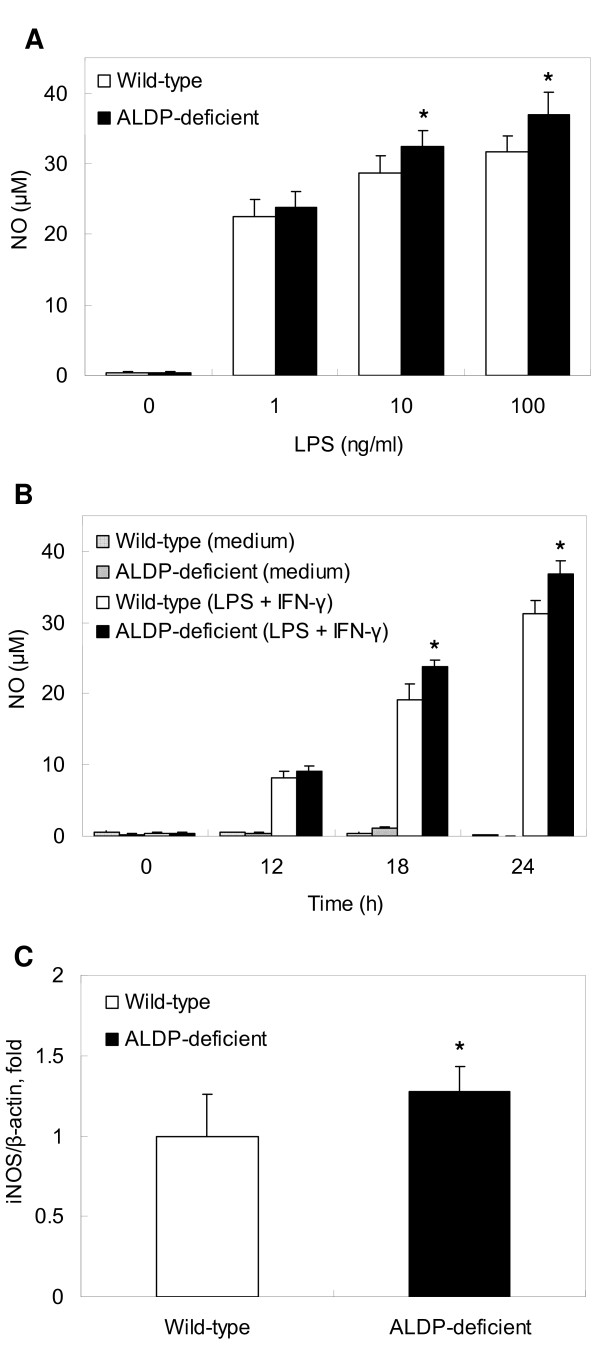
**NO production and iNOS gene expression in macrophages**. (A) Cells were incubated with LPS (1, 10, 100 ng/ml) plus IFN-γ (2 ng/ml) for 24 h at 37°C. Control contained neither LPS nor IFN-γ. After incubation, culture supernatants were collected and NO was measured with Griess reagent. (B) Cells were incubated with LPS (10 ng/ml) plus IFN-γ (2 ng/ml) for indicated period at 37°C. After incubation, culture supernatants were collected and NO was measured with Griess reagent. Results are expressed as mean ± SD, n = 6. (C) Cells were incubated with LPS (10 ng/ml) plus IFN-γ (2 ng/ml) for 18 h at 37°C. After incubation, total RNA was extracted and iNOS gene expression was analyzed by real-time RT-PCR. Results are mean-fold increase in iNOS mRNA levels of MPMs from ALDP-deficient mice compared with those of wild-type mice and expressed as mean ± SD, n = 6. * *P *< 0.05 for comparison with wild-type.

As shown in Fig. 2A, increased NO production of MPMs from ALDP-deficient mice was observed at medium (*P *< 0.05) and high (*P *< 0.05) doses compared with that of wild-type mice. As shown in Fig. 2B, NO production in MPMs from ALDP-deficient mice was significantly higher compared with those from wild-type mice at 18 h and 24 h (both *P *< 0.01). Fig. 2C shows iNOS gene expression. iNOS gene expression was significantly higher in MPMs from ALDP-deficient mice than in those from wild-type mice (*P *< 0.05).

### Intracellular ROS Production

**Figure 3:**
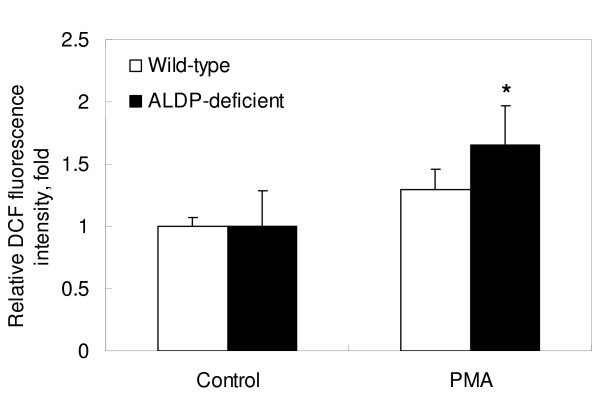
**Intracellular ROS productions in macrophages**. Cells were incubated with 10 μM of DCFH-DA for 15 min, and then stimulated with 0.5 μg/ml of PMA for 20 min at 37°C. Fluorescence of DCF was measured by a flow cytometer as described in Methods. Values are mean-fold increase of relative DCF fluorescence intensity compared with non-stimulated control and expressed as mean ± SD, n = 6. * *P *< 0.05 for comparison with wild-type.

Fig. 3 shows that intracellular ROS production levels were significantly higher in MPMs from ALDP-deficient mice than in those of wild-type mice (*P *< 0.05).

### Pro-Inflammatory Cytokine Production

**Figure 4:**
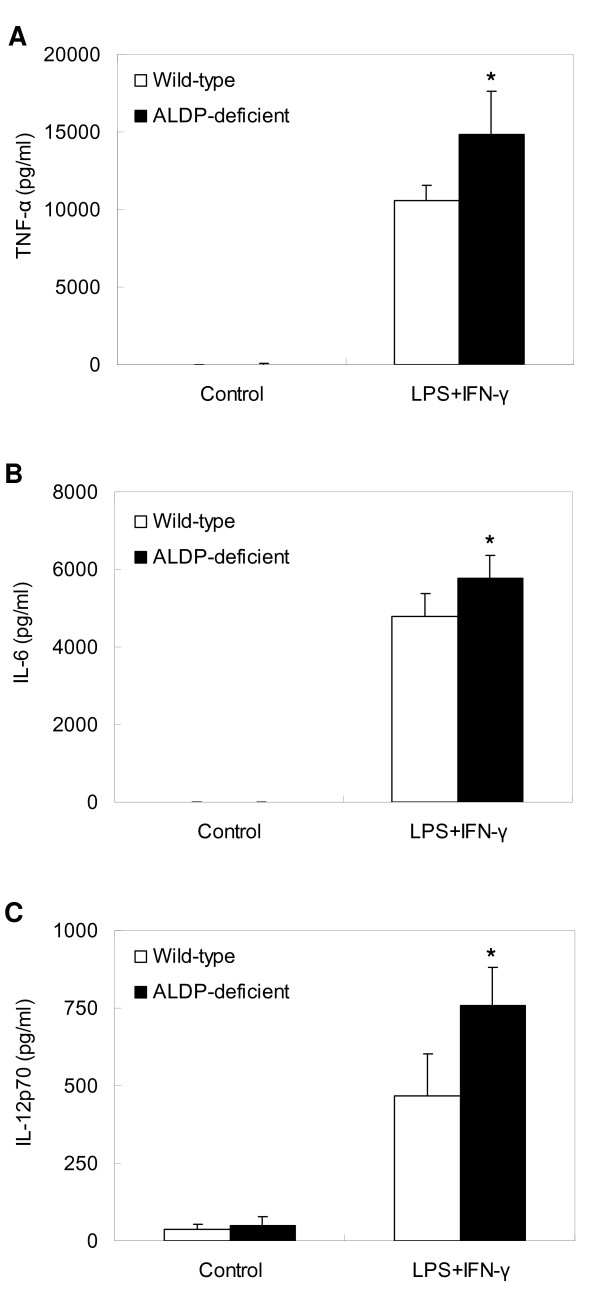
**Pro-inflammatory cytokine production in macrophages**. Cells were incubated with LPS (10 ng/ml) plus IFN-γ (2 ng/ml) for 24 h at 37°C. After incubation, culture supernatants were collected and TNF-α (A), IL-6 (B), and IL-12p70 (C) were measured by cytometric bead assay method as described in Methods. Results are expressed as mean ± SD, n = 6. * *P *< 0.05 for comparison with wild-type.

As shown in Fig. 4, the production of TNF-α, IL-6, and IL-12p70 in MPMs from ALDP-deficient mice was significantly higher than in those from wild-type mice after treatment with LPS and IFN-γ for 24 h (all, *P *< 0.05).

## Discussion

The present study firstly demonstrated that 1) macrophages from ALDP-deficient mice had significantly higher levels of VLCSFA, such as C24:0 and C26:0, compared with those from wild-type mice, 2) VLCSFA-accumulated macrophages had significantly higher production of inducible NO and pro-inflammatory cytokines, 3) VLCSFA-accumulated macrophages also exhibited significantly higher levels of intracellular ROS production compared with those of wild-type mice. These results suggested that VLCSFA accumulation in macrophages might contribute to pathogenesis of inflammatory diseases through the enhancement of inflammatory and oxidative responses.

The present study showed that VLCSFA-accumulated macrophages from ALDP-deficient mice had significantly increased NO and intracellular ROS production compared with macrophages from wild-type mice. These results were concordant with a previous study in which C26:0-enriched C6 glial cells had increased production of NO and O_2_^- ^after treatment with LPS or oxLDL [13]. Another study reported that C24:0 as well as C26:0 activated NADPH oxidase in human dermal fibroblasts [26]. Our study also confirmed high levels of iNOS mRNA expressions in macrophages from ALDP-deficient mice. Treatment with LPS and pro-inflammatory cytokines, such as TNF-α and IFN-γ, stimulates iNOS expression by activating nuclear factor-kappa B (NF-κB) in macrophages [27,28]. Although the precise mechanism of the intracellular pathway is still unknown, our results suggest that VLCSFA accumulation in macrophages may induce NF-κB activity after LPS and IFN-γ treatment. It has been reported that excess production of NO followed by activated iNOS influence the pathogenesis of inflammatory diseases, such as atherosclerosis. High levels of NO induce apoptosis of vascular smooth muscle cell through enhancing Fas/Fas-L interactions, which promote plaque instabilities [29,30]. In addition, interaction of NO and O_2_^- ^rapidly generates peroxynitrite (ONOO^-^), which has injurious effects on vascular cells through modification of lipid, protein, and DNA [31-33]. As we recently reported high levels of C26:0 and C24:0 in red blood cells of patients with coronary artery disease and metabolic syndrome, VLCSFA-accumulated macrophages might contribute to the initiation and progression of atherosclerosis through the enhanced production of NO and ROS.

The production of pro-inflammatory cytokines was increased in macrophages from ALDP-deficient mice. These findings were consistent with previous studies using C26:0-enriched C6 glial cells [13] and PBMC from X-ALD patients [11,12]. In the present study, we firstly demonstrated not only enhanced production of a variety of pro-inflammatory cytokines, but also the accumulation of VLCSFA in detail, utilizing macrophages from ALDP-deficient mice. The increased levels of VLCSFA, such as C24:0 and C26:0, were clearly observed in macrophages from the ALDP-deficient mice. Although the relative percentages of total n-6 PUFA or n-3 PUFA, including eicosapentaenoic acid (EPA: C20:5n-3) and docosahexaenoic acid (DHA: C22:6n-3), were not significantly different between the two groups, the ratio of n-3/n-6 was significantly deceased in macrophages from the ALDP-deficient mice compared with those from wild-type mice. The feature of susceptibility to inflammatory and oxidative responses in macrophages from the ALDP-deficient mice could derive from the low ratio of n-3/n-6; however, the ratio of n-3/arachidonic acid (AA: C20:4n-6) was similar between the two groups. Therefore, we believe that the findings of the present study were caused mainly by VLCSFA accumulation in macrophages.

Although we have not confirmed that ALDP-deficient mice have the features of metabolic syndrome and/or coronary artery disease compared to wild-type mice, body weight was higher in ALDP-deficient mice than wild-type mice. A previous study reported that plasma TC levels were elevated in the ALDP-deficient mice [34]; however, the levels of TC, TG, LDL-C or HDL-C, were not significantly different between the ALDP-deficient mice and the wild-type mice in the present study. Further analysis is needed to clarify the mechanism behind differences of body weight between the two groups.

## Conclusion

VLCSFA-accumulated macrophages show enhanced production of NO, ROS, and pro-inflammatory cytokines, suggesting that VLCSFA accumulation in macrophages may contribute to the pathogenesis of inflammatory diseases through the enhancement of inflammatory and oxidative responses. Further studies are needed to investigate the direct evidence for VLCSFA accumulation inducing inflammatory disease such as coronary artery disease.

## Competing interests

The authors declare that they have no competing interests.

## Authors' contributions

NY participated in planning of the study, experimental work, analysis and publication of results. KS contributed to the design of the study, data analysis, and prepared the manuscript. TM, AK, and TK participated in the planning of the study and discussion of results. YK participated in lipid analysis. KS and TI contributed to experimental work and discussion of results. NI was involved in data analysis and discussion of results. HD contributed to the planning of the experiment and discussion of results, and supervised the study.
